# Whole-Body Physiologically Based Pharmacokinetic–Pharmacodynamic Modeling for Interspecies Translation and Mechanistic Characterization of Plasma and Tissue Disposition of GalNAc-siRNAs

**DOI:** 10.3390/pharmaceutics17091154

**Published:** 2025-09-03

**Authors:** Emilie Langeskov Salim, Kim Kristensen, Girish Chopda, Erik Sjögren

**Affiliations:** 1 Department of Pharmaceutical Bioscience, Translational Drug Discovery and Development, Uppsala University, SE-75124 Uppsala, Sweden; 2 Department of Discovery PKPD & QSP Modelling, Novo Nordisk A/S, DK-2760 Måløv, Denmark; 3 Department of Nonclinical and Clinical Pharmacology, Novo Nordisk, 800 Scudders Mill Road, Plainsboro, NJ 08536, USA

**Keywords:** GalNAc-siRNA, physiologically based pharmacokinetic modeling, PK-Sim, species translation

## Abstract

**Introduction/aim:** N-acetylgalactoseamine-conjugated small interfering RNAs (GalNAc-siRNAs) are an emerging class of drugs possessing an extensive clinical potential because of their high target specificity to the asialoglycoprotein receptor (ASGPR) in hepatocytes. Overall, GalNAc-sRNAs are well-tolerated across species but differences in pharmacokinetic (PK) and pharmacodynamic (PD) properties have been observed. Furthermore, despite GalNAc-siRNA’s high liver specificity, distribution into off-target organs does occur. Through whole-body physiologically based pharmacokinetic (PBPK) modeling, this study seeks to mechanistically address species differences, establish clinical PK-PD relationships, and characterize off-target organ accumulation, ultimately expediting the preclinical-to-clinical translation of GalNAc-sRNAs in drug development. **Materials/Methods:** For model development, validation, and establishment of species’ translations, three in-house GalNAc-siRNAs with PK data from different biospecimens, as well as downstream effects on mRNA and target proteins in mouse, monkey, and human, were leveraged. A WB-PBPK-PD legacy model, developed as an extension to the generic model for large molecules in the platform Open Systems Pharmacology Suite, was further validated and applied to address the specific aims of this study. **Results:** The model successfully quantified the PK-PD relationships across species and characterized accumulation in off-target organs. The model further sheds light on species-specific differences, such as liver permeability, subcutaneous absorption rate, as well as PD-related mechanisms. Moreover, the model confirmed previously established compound-specific pharmacokinetic differences and similarities. **Conclusions**: This PBPK-PD can serve as a framework for future investigations of novel GalNAc-siRNAs across species.

## 1. Introduction

Small interfering RNAs (siRNAs) are a type of oligonucleotide that plays a crucial role in endogenous gene regulation on a post-transcriptional level. siRNAs consist of a sense strand and an antisense strand, in which the latter activates RNA interference pathways promoting mRNA silencing and preventing protein translation. The mRNA silencing occurs in the cell cytosol, where the antisense strand loads into a family of Argonaut 2 proteins (Ago2) and forms the RNA-induced silencing complex (RISC). The RISC catalytically induces mRNA degradation with the antisense strand as guidance to its complementary mRNA, which prevents protein translation [1]. Interest in siRNAs as a therapeutic modality has grown significantly over the past two decades [2]. However, the physicochemical properties of unmodified siRNAs limit their clinical potential as they have a high molecular weight (13–22 kDa), are vulnerable to nuclease activity in plasma, and they possess a negative net charge, which limits passive cell membrane translocation.

Different chemical modifications of the siRNA backbone, such as enhanced chemical stabilization (ECS), advanced ECS (Adv ECS), and tetra-hairpin loop designs, are well-documented modifications that improve the drugability of siRNAs [3,4]. Combined with chemical modifications of the siRNA, direct conjugation of the multivalent N-Acetyl-Galactose-Amine (GalNAc) onto the siRNA (GalNAc-siRNA) is the most successful delivery strategy. The GalNAc linker specifically targets the asialoglycoprotein receptor (ASGPR), abundantly expressed on hepatocytes [5].

The target specificity of the GalNAc-siRNA to ASGPR leads to rapid distribution and high accumulation in the liver as well as fast disappearance from plasma [5,6,7]. Once the GalNAc-siRNA is translocated by the ASGPR to the endosome, the more acidic environment promotes a spontaneous GalNAc-siRNA sequestration and the ASGPR returns to the cell surface. Approximately ≤1% of the siRNA continuously escapes into the cell cytoplasm from the endosome for subsequent loading into the RISC [8]. Despite high target specificity of the GalNAc ligand to the liver, other GalNAc-conjugated oligonucleotides have shown distribution to off-target tissues and recirculation into plasma via unknown mechanisms, including GalNAc-siRNA [9,10]. The unique pharmacokinetic and pharmacodynamic characteristics of GalNAc-siRNAs—including their general tissue distribution and recirculation, short plasma half-life, high liver specificity, and prolonged downstream effects on target proteins—make multiscale modeling approaches like physiologically based pharmacokinetic (PBPK) models appropriate for characterizing their pharmacokinetic–pharmacodynamic (PK-PD) relationship. Furthermore, as PBPK models are built upon the anatomical and physiological characteristics of the organism as well as the properties of the drug, they are exceptionally well-suited for mechanism-based translations across species.

Our group recently presented a Whole-Body PBPK-PD (WB-PBPK-PD) mouse model [11], leveraging the established model for large molecules, i.e., mAbs, in open source software PK-SIM ^®^/MOBI ^®^ version 11.2 [(https://www.open-systems-pharmacology.org/)] (accessed on 1 February 2025) in mouse with the aim of mechanistically explaining the extravasation of GalNAc-siRNA via the two-pore formalism theory [12] and individually representing each organ. The previously published WB-PBPK-PD model was developed and evaluated using data from plasma, liver, kidney, and RISC formation but not including data for off-target organ exposure or the redistribution to plasma. To our knowledge, this is the first time that general organ distribution data for GalNAc-siRNAs, along with a corresponding mechanistic model, have been presented [9,10].

The first aim of this study was to further validate the previously published model by Salim et al. 2025 [11] with respect to quantifying general organ disposition and plasma. The second aim of this study was to leverage these new insights to inform mechanism-based species translations, from mice into higher-order species, for the establishment of clinical PK-PD relationships, and to explore possibilities for preclinical-to-clinical translations in drug development of GalNAc-siRNAs.

## 2. Materials and Methods

### 2.1. Data Collection

To validate a previously published WB-PBPK-PD model, a dataset from three different studies on GalNAc-siRNAs (siRNA-1, siRNA-2, and siRNA-3) with different target proteins, all formulated with a tetra-hairpin loop stabilization design for subcutaneous administration in mouse, was collected. The original purpose of the trials was to quantify the concentration in plasma, liver, kidney, and other tissues (spleen, heart, brain, and lung). All procedures involving mice were reviewed by UL Lafayette’s Animal Care and Use Committee (IACUC) prior to the initiation (AAALAC Number: 000452). All the mice included in the analysis were acquired from Charles River Laboratories and administered doses of 3, 10, 100, or 300 mg/kg. In each of the three studies, a total of 180 male CD-1 mice (age 6 to 8 weeks) were included. The mice were randomly assigned to study groups (*n* = 3 per cage), and terminal blood and tissues were collected at warranted time points (*n* = 3 per time point). Only siRNA-1 had available liver mRNA data to validate the PD implementation. Further details on the available mouse data are summarized in Table 1.

Pharmacokinetic (PK) species translation and clinical PK-PD relationships were established using data on siRNA-1, siRNA-2, and siRNA-3 in monkey and human after subcutaneous administration. Similar to the mouse studies, all procedures involving monkey were reviewed by UL Lafayette’s Animal Care and Use Committee (IACUC) prior to the initiation (AAALAC Number: 000452). To characterize the PK-PD relationship in monkey, data from three distinct trials in cynomolgus monkeys were collected. The trials were formerly conducted to evaluate PK and PD properties. For siRNA-2 and siRNA-3, 5 cynomolgus monkeys, mixed sex, were assigned to each study and administered a dose of 1 mg/kg and 3 mg/kg, respectively. Terminal blood and liver biopsies were collected at warranted time points. For siRNA-1, a total of 8 cynomolgus monkeys, mixed sex, were included and randomly assigned into two study groups, receiving either 1.5 mg/kg or 3 mg/kg. The monkey data included measurements of plasma concentrations, liver tissue concentrations, and liver mRNA in doses of 1–3 mg/kg for all three compounds. Table 1 summarizes the available monkey data.

To assess the clinical PK-PD relationship, mean data from three individual randomized, single- or double-blind, placebo-controlled phase I studies involving healthy volunteers were derived. The study protocol was designed jointly by the investigators and the sponsor (Dicerna Pharmaceuticals, Inc., Lexington, MA, USA), and approved by institutional review boards or local ethics committees. The studies were conducted according to the provisions of the Declaration of Helsinki and Good Clinical Practice Guidelines of the International Conference on Harmonisation. These trials were primarily designed to evaluate the safety, tolerability, PK, and/or PD of increasing single doses. For siRNA-1, 24 healthy volunteers were assigned into 4 dose cohorts (1–13 mg/kg), (*n* = 4 active, *n* = 2 placebo, in each dose cohort); for siRNA-2, 30 healthy volunteers were assigned into 5 dose cohorts (*n* = 4 active, *n* = 2 placebo, in each dose cohort); and for siRNA-3, 25 healthy volunteers were assigned into 5 dose cohorts (1.5–12 mg/kg) (*n* = 2 active, *n* = 3 placebo, in each dose cohort). For further details on siRNA-1, siRNA-2, and siRNA-3, see clinicaltrials.gov: NCT05021640, NCT04174118, and NCT03392896, respectively. The human trials included data on plasma concentrations from every dose cohort, while downstream effects on target proteins were only available for siRNA-2 (4 dose cohorts, 0.1–6 mg/kg) and siRNA-3 (2 dose cohorts, 3–6 mg/kg). Moreover, the monkey and the human model was further explored with a compound from the literature, Olpasiran©, including plasma concentrations and downstream effects of target protein in doses of 10 mg/kg for monkey and doses of 3–225 mg in human [13]. Further details on the available monkey and human data are summarized in Table 1. A tissue density of 1 mg/mL was assumed for converting the liver and kidney tissue concentrations from weight-based to volume-based measurements.

### 2.2. Software

The WB-PBPK-PD model presented by Salim et al., 2025 [11] was applied in this study. This model was developed and validated as an extension to the generic model for proteins and large molecules implemented in the open-source platform Open Systems Pharmacology Suite, PK-Sim^®^/MoBi^®^, Version 11.2 [(https://www.open-systems-pharmacology.org/)] (accessed on 1 February 2025) [12]. PK-parameter calculations and creation of visualizations were performed via the R packages ncappc and ggplot2, respectively, CRAN package ncappc: 10.32614/CRAN.package.ncappc and CRAN package ggplot2: 10.32614/CRAN.package.ggplot2. https://www.open-systems-pharmacology.org/)] (accessed on 3 March 2025) Final WB-PBPK-PD model simulations and sensitivity analyses were performed applying the ospsuite R package v12.2.0 (https://github.com/Open-Systems-Pharmacology/OSPSuite-R/releases/tag/v12.2.0) (accessed on 23 August 2025). All R activities were performed via R Studio (Version: 2025.05.1+513) [RStudio Team (2023). RStudio: Integrated Development Environment for R. RStudio, PBC. http://www.rstudio.com/] (accessed on 1 March 2025) [14]. The literature data on Olpasiran© was extracted via digitization (WebPlotDigitizer, version 4.6; https://automeris.io).

### 2.3. General Model Development and Assumptions

The general model structure included 15 individually represented tissue compartments divided into 4 subcompartments representing the vascular space, interstitial space, endothelial endosomes, and the intracellular space all connected by organ-specific blood and lymph flows. The extravasation from vascular endothelial endosomes in the different tissue compartments was characterized via generic implementation of the two-pore formalism. The two-pore formalism describes extravasation in each tissue via an organ-specific flux rate dependent on the permeability, capillary surface areas, etc., as well as compound-specific parameters [12]. In addition to the two-pore formalism, the extravasation of GalNAc-siRNAs in the liver also involved bi-directional translocation via passive permeability [11]. The general tissue and organ distribution was further described by first-order uptake and redistribution rates (k_uptake.tissue_ and k_recycle.tissue_) from the interstitial and endothelial–endosomal compartments, while intracellular uptake was set to zero. Renal tissue elimination was described via glomerular filtration of unbound drug in plasma, parameterized according to the physiological database. Potential RNase activity was implemented as a first-order metabolic reaction in plasma and in the endosomal compartment of the kidney and additional tissues and organs while being disabled in the liver. The RNase activity in the liver was modeled independently from additional tissue compartments as a first-order degradation rate in the liver endosomes, denoted as kendosome. Figure S1 summarizes the full model’s structure.

The liver dynamics and ASGPR-mediated uptake were further characterized with an extended target-mediated drug disposition (TMDD) model implemented in MoBi^®^. The TMDD model leveraged on the original TMDD model developed by Krzyzanski et al. [14] that was further extended by Ayyar et al., 2021 [15]. Initial model parameter values were informed by compound characteristics, the default settings in PK-Sim^®^, and as reported in the ASGPR studies [11,15]. A “middle-out approach”, combining in vivo/in vitro information with optimization of model parameters, was adopted for each step in the model development and species translation. Parameter optimization was performed using the Monte Carlo simulation optimization algorithm available in MoBi^®^ with default settings to break condition for relative error improvement (0.001). All parameter estimates were also assessed in terms of biological plausibility. For each step in the model development, an iterative strategy was adapted, comparing simulation outputs to the reference data. For further details on general model assumptions, see Salim et al. 2025 [11].

### 2.4. Model Validation

Initially, the legacy model [11] was validated against data obtained for siRNA-1, siRNA-2, and siRNA-3 in mouse studies. The majority of globally estimated PBPK parameters were fixed during this model validation in accordance with the legacy WB-PBPK-PD model, i.e., the ASGPR-GalNAc-siRNA complex formation, internalization, and receptor density. However, as highlighted in the legacy model report, some compound differences were to be expected [11]. Different chemical stabilization designs may contribute to differences in RNase-mediated degradation and unspecific endosomal tissue distribution as well as endosomal stability in the liver [6,11,16]. Therefore, the endosomal degradation rate (k_endosome_), mainly governing the prolonged half-life in the liver, was estimated for siRNA-1, siRNA-2, and siRNA-3 during model validation. Finally, due to new data informing distribution to additional organs (heart, lung, gonads, and spleen) and redistribution to plasma, re-estimation of k_uptake.tissue_, k_recycle.tissue_, and RNase activity was possible. Figure S2 shows a schematic representation of the modeling strategy in which data were utilized in the different modeling steps.

### 2.5. Species Translation

After model validation, the model was scaled to higher-order species—monkey and human—with the purpose of establishing a clinical PK-PD relationship and the foundation for species translations. Initially, a monkey and a human WB-PBPK-PD model were established based on the physiological and anatomical database provided in PK-Sim^®^ to consider these species differences. The monkey and the human model were evaluated against reference data (Table 1), with the estimates obtained from the mouse model validation. A majority of the parameter estimates for mouse were directly adopted for the monkey and the human model. However, for the models to accurately describe observations, a few parameters required further refinement, as described below. The endosomal degradation rate in the liver (k_endosome_) was scaled allometrically by body weight for each species using a scaling exponent of −0.25 (Equation (1))

(1) $$k_{endosome_{i}}=k_{endosme_{std}}\times {\left(\frac{BW_{i}}{BW_{std}}\right)}^{-0.25}$$

where k_endosme,std_ represents the k_endosme_ in a standardized individual with bodyweight BW_std_; and k_endosome,i_ represents the k_endosome_ in the individual i with body weight BW_i_.

The remaining parameters requiring species-specific calibration, i.e., ka, liver permeability (P_liver_), k_liver.recycle_, k_DR_, and k_onRISC_, were estimated via Monte Carlo simulations for both the monkey and the human model. The receptor density of ASGPR (R_tot_) was scaled in accordance with the previous scaling method applied in an mPBPK-PD model published by Ayyar et al. 2021 [15]. For the full model overview, see Supplementary Materials Model File: Case_Example_WB-PBPK-PD_Model_Human_3_mgkg_siRNA-2.

### 2.6. PK-PD Relationship

The PK-PD relationship was described in accordance with the legacy model [11] as the relative change to baseline in endogenous mRNA via an indirect response model as given in Equation (2):

(2) $$\frac{dmRNA}{dt}=k_{deg.mRNA}\times \left(mRNA_{0}-\left(1+\frac{S_{max}\times CRISC}{SC_{50}+CRISC}\right)\times mRNA\right)$$

where k_deg.mRNA_ represents the degradation rate constant of the target mRNA; mRNA_0_ is the baseline value of mRNA equal to 1; CRISC is the concentration of the siRNA-induced RISC; S_max_ describes the maximum effect induced; and SC_50_ is the concentration required to produce half the maximum effect. The knockdown of target protein was modeled into the mRNA level relative to the mRNA baseline (mRNA_0_), as shown in Equation (3):

(3) $$\frac{dProtein}{dt}=k_{deg.protein}\times \left(Protein_{0}\times {\left(\frac{mRNA}{mRNA_{0}}\right)}^{y}-Protein\right)$$

where k_deg.protein_ represents the degradation rate constant of the target protein; and γ is an empirical power function describing the slope of the response to the change in mRNA levels. The absolute value for mRNA_0_ and Protein_0_ was set to 100%.

## 3. Model Evaluation

### 3.1. Sensitivity Analysis

To identify PBPK-specific sensitive parameters, a local sensitivity analysis on changes to the AUC_0-8000h_ was performed on the human model. The sensitivity of each PK parameter was calculated as the ratio of the relative change in the AUC_0-8000h_. The relative variation in the input parameter, i.e., the perturbation, was explored with a variation of 10% with sensitivity coefficients calculated as in Equation (4):

(4) $$S_{ij}=\frac{∆PK_{j}}{∆p_{i}}\times \frac{p_{i}}{PK_{j}}$$

where ∆PK_j_ is the change in the PK parameter; ∆p_i_ is the change in the input parameter; PK_j_ is the value of the PK parameter; and p_i_ is the value of the input parameter. The sensitivity coefficients were evaluated according to WHO guidelines as being high (absolute value ≥ 0.5), medium (absolute value ≥ 0.2 but less than 0.5), or low (absolute value ≥ 0.1 but < 0.2) [17].

### 3.2. Model Performance

The overall model performance was evaluated quantitatively by calculating the geometric mean of the ratio between the absolute simulated area under the curve (AUC_sim_) and the observed area under the curve (AUC_obs_) estimate as the absolute average fold error (AAFE), along with the average fold error (AFE). Further details on the model performance assessment are summarized in the Supplementary Materials. Given the variability and sparsity of the compiled reference data, the model was considered to perform adequately when AFE values were between 0.5 and 2 (0.5 ≤ AFE ≤ 2) and AAFE values were 2 or less (AAFE ≤ 2).

## 4. Results

### 4.1. Model Validation and Characterization of General Tissue Distribution

**Figure 1:**
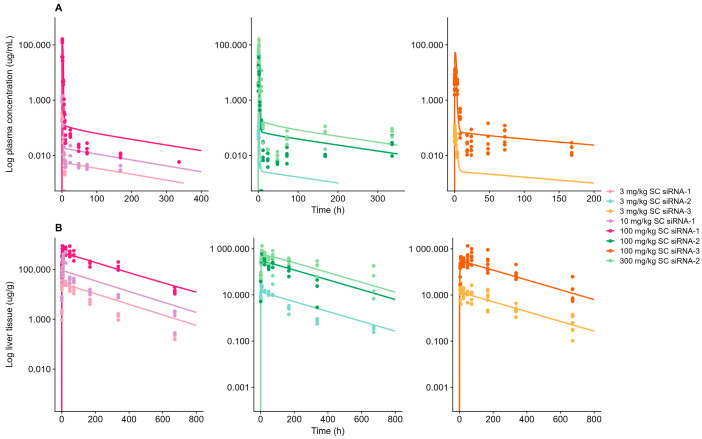
Model-simulation vs. observed data for subcutaneous (SC)-administered GalNAc-conjugated siRNA-1, siRNA-2, and siRNA-3 in mouse: (**A**) Plasma concentrations. (**B**) Liver tissue concentrations. Solid line represents model-simulated concentrations, and dots represent observed data. Left panels show data on siRNA-1 after 3 mg/kg (light pink), 10 mg/kg (light purple), and 100 mg/kg (dark pink). Mid panel shows siRNA-2 after 3 mg/kg (light blue), 100 mg/kg (dark green), and 300 mg/kg (light green). Right panel shows siRNA-3 after 3 mg/kg (light orange) and 100 mg/kg (dark orange).

The legacy model predicted the target tissue (liver) disposition of siRNA-1, siRNA-2, and siRNA-3 in mice by applying the reported ASGPR-related model parameters (Figure 1B) from the legacy model. AFE and AFFE in predicted liver tissue exposure were 0.78 and 1.39, with liver tissue concentrations predicted within a 2-fold deviation to observations. The predicted kidney tissue exposure was well-characterized (Figure S3) by 2-fold, using the legacy model estimates for endosomal RNase abundance: 1.17 µmol/L and k_kid.recycle_: 3.91 × 10^−4^ min^−1^, and re-estimation of the unspecific endosomal uptake k_uptake.tissue_ (25 min^−1^). The AFE and AFFE of the kidney exposure met the criteria for adequate model performance with values of 0.97 and 1.53, respectively.

Optimization of endosomal degradation rate (k_endosome_) in the liver was necessary to improve model performance, in accordance with expectations related to stabilization design and RNA sequence dependencies [11]. Adequate model performance was achieved for the in-house compounds with a common estimate for k_endosome_ of 5.0 × 10^−3^ h^−1^. However, compound-specific estimates of subcutaneous bioavailability (F) were identified to improve the ability of the model to capture the observed data: F = 22% for siRNA-2 and siRNA-3; F = 47% for siRNA-1. Model parameters are summarized in Table 2 and Table 3. The plasma concentration profiles for all investigated siRNAs displayed three phases, indicated by an absorption phase, a rapid elimination/distribution phase, and a third redistribution phase, starting approximately at 10 h, with a long terminal half-life. The plasma redistribution was mainly assumed to originate from slow systemic redistribution from tissue back into plasma. In the legacy model, this slow redistribution was integrated for all off-target organs [11]. Nonetheless, the amount of drug distributed to off-target tissues, i.e., amount available for redistribution, was not sufficient to describe observations beyond 10 h. To address the long terminal half-life in plasma, unspecific endosomal uptake/recycling rates were incorporated into the liver tissue, enabling an increased amount of drug to be redistributed into plasma. The unspecific endosomal trafficking in liver was described by the generic implementation of the platform for endosomal uptake (k_uptake.liver_ = 0.29 min^−1^). The endosomal recycling rate, k_recycle.liver_, was estimated to be 1.33 × 10^−3^ min^−1^. With the incorporation of redistribution from the liver, the model was able to capture the three-phase plasma PK within a 2-fold range of the observed data for most of the cases (Figure 1A). Furthermore, the AFE and AFFE were estimated at 1.48 and 1.63, respectively, for the predicted plasma exposure, suggesting a slight trend of overprediction, while remaining within the requirements for satisfactory model performance. The subcutaneous absorption was successfully characterized by maintaining the subcutaneous absorption rate to the value calculated in the legacy model (0.84 h^−1^).

**Figure 2:**
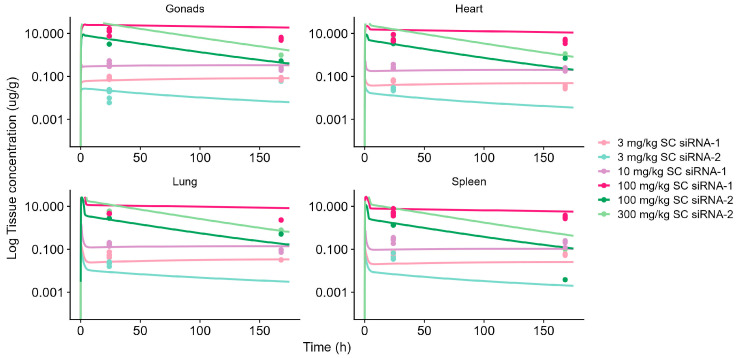
Model-simulated tissue concentration profiles in gonads, heart, lung, and spleen vs. observed data for subcutaneous (SC)-administered GalNAc-conjugated siRNA-1 and siRNA-2 in mouse. Solid lines represent model-simulated concentrations and dots represent observed data. Light pink line represents siRNA-1, 3 mg/kg (SC). Light blue line represents siRNA-2, 3 mg/kg (SC). Purple line represents siRNA-1, 10 mg/kg (SC). Dark pink line represents siRNA-1, 100 mg/kg (SC). Dark green line represents siRNA-2, 100 mg/kg (SC). Light green line represents siRNA-2, 300 mg/kg (SC).

The model accurately predicted the accumulation into off-target tissues (lung, heart, spleen, and gonads), illustrated in Figure 2. Extravasation in off-target tissues was characterized using the two-pore formalism, while tissue distribution was characterized by non-specific endosomal trafficking defined by an uptake rate (k_uptake.tissue_) and a recycling rate (k_recycle.tissue_). The k_uptake.tissue_ was estimated as a tissue-specific parameter with gonads showing the highest k_uptake.tissue_ of 1.48 min^−1^, then heart (0.38 min^−1^), spleen (0.17 min^−1^), and lung (0.06 min^−1^). To avoid model overparameterization, k_uptake.tissue_ into remaining tissues was fixed to the generic value provided by the platform (0.29 min^−1^). The k_recycle.tissue_ in off-target tissues was estimated, as a compound-specific parameter, to be 3.23 × 10^−5^ min^−1^ for siRNA-1 and siRNA-3 and to be 3.49 × 10^−4^ min^−1^ for siRNA-2. To adequately account for the long half-life in off-target tissues, the previously established RNase elimination rate in the legacy model was maintained but RNase abundance was re-estimated to be 2.75 × 10^−2^. All model parameters describing off-target distribution are summarized in Table 2 and Table 3.

The PK-PD relationship was validated against target mRNA for siRNA-1. Optimization of the RISC complex degradation rate (k_DR_), Smax, and SC_50_ was deemed necessary to successfully describe the PK-PD relationship. The model identified a k_DR_ of 9.72 × 10^−3^ h^−1^, S_max_ of 58.83, and SC_50_ of 3.52 nmol/L. Following the optimization of the model parameters, the model successfully characterized the mRNA knockdown across doses, as shown in Figure S4.

### 4.2. Model Species Translation

**Figure 3:**
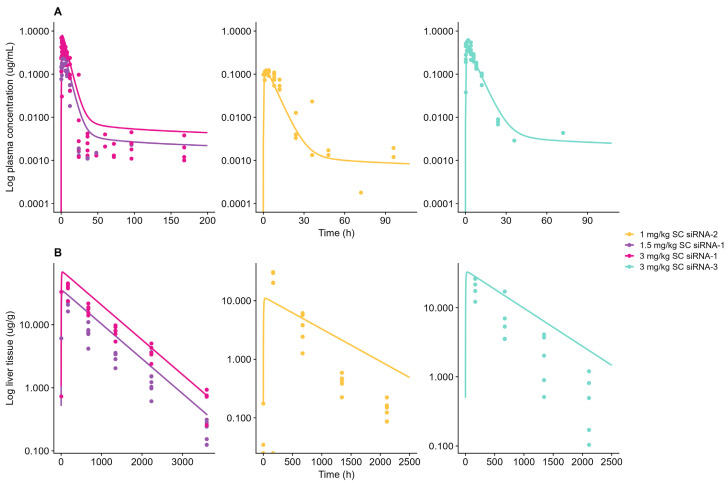
Model-simulation vs. observed data for subcutaneous (SC)-administered GalNAc-conjugated siRNA-1, siRNA-2, and siRNA-3 in monkey: (**A**) Plasma concentrations. (**B**) Liver tissue concentrations. Solid line represents model-simulated concentrations, and dots represent observed data. Left panels show siRNA-1 after 1.5 mg/kg (dark purple) and 3 mg/kg (dark pink). Mid panels show siRNA-2 after 1 mg/kg (yellow). Right panels show siRNA-3 after 3 mg/kg (light blue).

**Figure 4:**
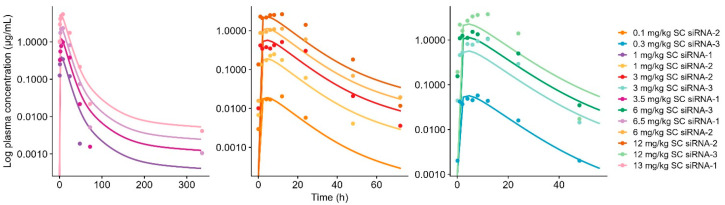
Model-simulated plasma concentration profiles vs. observed data for subcutaneous (SC)-administered GalNAc-conjugated siRNA-1, siRNA-2, and siRNA-3 in human. Solid line represents model-simulated concentrations, and dots represent observed data. Left panel shows data on siRNA-1 after 1 mg/kg (dark purple), 3.5 mg/kg (dark pink), 6.5 mg/kg (light purple), and 13 mg/kg (dark pink). Mid panel shows siRNA-2 after 0.1 mg/kg (orange), 1.0 mg/kg (yellow), 3 mg/kg (red), 6 mg/kg (light orange), and 12 mg/kg (brown). Right panel shows siRNA-3 after 0.3 mg/kg (blue), 3 mg/kg (light blue), 6 mg/kg (dark green), and 12 mg/kg (light green).

The mouse WB-PBPK-PD model was scaled to monkey and human by adopting the species translation strategies as outlined in the Materials and Methods Section. Both models successfully captured respective reference plasma concentration data (Figure 3A and Figure 4).

P_liver_, ka, and k_recycle.liver_ was optimized to 1.56 × 10^−3^ cm/min, 4.22 h^−1^, and 2.30 × 10^−5^ h^−1^, respectively, for the monkey model. The appropriateness of the systemic parameterization was further supported by adequate description of a 10 mg/kg SC dose Olpasiran© applying final systemic parameter values (Figure S5A). Compound-specific model parameters such as F and k_recycle.tissue_ were obtained from siRNA-3.

Plasma data on Olpasiran© were included in addition to sRNA-1, siRNA-2, and siRNA-3 in the establishment of the human model to facilitate identification of the plasma redistribution process. For the human model, P_liver_, ka, and k_recycle.liver_ was optimized to 1.21 × 10^−5^ cm/min, 7.18 h^−1^, and 4.27 × 10^−5^ h^−1^, respectively. Human plasma simulations of Olpasiran© are shown in Figure S6A. Model-simulated plasma concentrations for both monkey and human were within a 2-fold of observations. The AFE and AFFE for plasma AUC were calculated as 0.85 and 1.39 for monkey and 0.62 and 1.61 for human, respectively, which indicates a minor trend of underprediction for both species. Furthermore, the monkey model successfully characterized liver tissue concentrations in (Figure 1B). The k_endosome_ was allometrically scaled on bodyweight with an exponent of −0.25, and the receptor density (R_tot_) was scaled to compensate for the decreased number of receptors per cell in higher-order species [16,19]. The liver tissue predictions in monkey were estimated within 2-fold compared to observed data, with an AFE of 1.32 and an AFFE of 1.41, suggesting a tendency toward a minor overprediction; however, both the AFE and AAFE met the criteria for successful model performance. No liver tissue data were available for humans to evaluate the appropriateness of the adopted scaling strategy or model performance in terms of describing liver exposure. Final model parameters for in-house compounds are summarized in Table 2, and final model parameters for Olpasiran© are summarized in Table S1.

### 4.3. Clinical PK-PD Relationship

**Figure 5:**
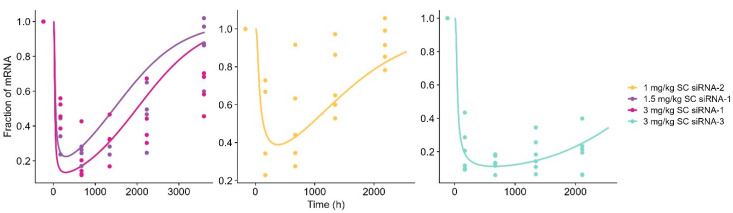
Model-simulated knockdown of target mRNA profiles vs. observed data for subcutaneous (SC)-administered GalNAc-conjugated siRNA-1, siRNA-2, and siRNA-3 in monkey. Solid line represents model-simulated concentrations, and dots represent observed data. The left panel shows siRNA-1 after 1.5 mg/kg (dark purple) and 3 mg/kg (dark pink). Mid panel shows siRNA-2 after 1 mg/kg (yellow). Right panel shows siRNA-3 after 3 mg/kg (light blue).

**Figure 6:**
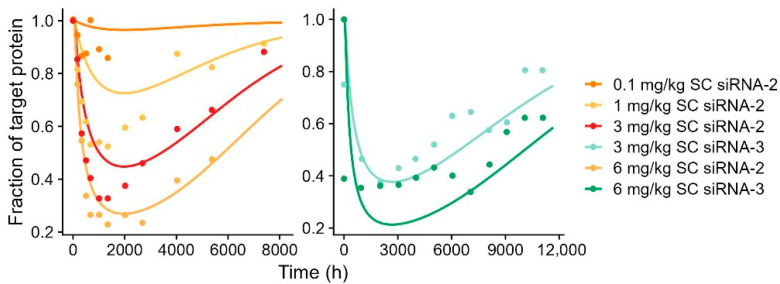
Model simulation of downstream effect on target protein vs. observed data for subcutaneous (SC)-administered GalNAc-conjugated siRNA-2 and siRNA-3 in human. Solid line represents model-simulated concentrations, and dots represent observed data. Left panel shows siRNA-2 after 0.1 mg/kg (orange), 1 mg/kg (yellow), 3 mg/kg (red), and 6 mg/kg (light orange). Right panel shows siRNA-3 after 3 mg/kg (light blue) and 6 mg/kg (green).

By identification of the compound and species-specific PD parameters, the model could describe the dynamics and maximum knockdown of target mRNA for monkey (Figure 5) and the downstream effect on target protein in human (Figure 6) with small adjustments (15–20%) to the maximum effect.

Due to a lack of reference data on siRNA-induced RISC formation in monkeys and humans, the model was first informed by RISC parameters and PD effect parameters identified for siRNA-1 in mice. However, to adequately describe the PK-PD relationship in monkeys and humans, some recalibration was required across compounds and species for the RISC parameters. The PD effect parameters were conserved across compounds and species. Furthermore, k_DR_ was optimized across compounds and species, while k_onRISC_ only required optimization in the human model. For siRNA-1, siRNA-2, and siRNA-3 in monkey, k_DR_ was identified to be 8.02 × 10^−3^ h^−1^, 5.28 × 10^−3^ h^−1^, and 1.94 × 10^−3^ h^−1^, respectively. For siRNA-2 and siRNA-3, the k_DR_ values in human were identified to be approximately 10-fold lower compared to the corresponding compound’s parameter value in monkey. The k_DR_ in humans was estimated to be 4.17 × 10^−4^ h^−1^ and 2.02 × 10^−4^ h^−1^ for siRNA-2 and siRNA-3, respectively. The k_onRISC_ in human was estimated as a common compound parameter to a value of 1.68 × 10^−5^ l/nmol/h, approximately 16-fold lower compared to k_onRISC_ in mouse and monkeys. The degradation rate of the liver mRNA (k_deg.mRNA_), target protein (k_deg.protein_), and the gamma (γ) were kept constant across all species, as previously reported [16]. All PD parameters are summarized in Table 2 and Table 3.

To further explore the species translation and the potential compound and species-specific differences in the establishment of the clinical PK-PD relationship, Olpasiran© in monkey and human was further explored. Initially, the PD effect parameters obtained from siRNA-3 in monkey were applied to Olpasiran© but optimization of the PD effect parameters and the k_DR_ was essential to characterize the downstream effect on the target protein. With optimized model parameters, the monkey model successfully characterized the downstream effect of target protein (Figure S5B) with an Smax of 36.4, SC50 of 8.61 nmol/l, and k_DR_ of 3.03 × 10^−3^ h^−1^. It was further attempted to describe the PK-PD relationship in human for Olpasiran© using the same strategy as for siRNA-1, siRNA-2, and siRNA-3 by applying the PD effect parameters identified for the monkey, with a 10-fold decrease in k_DR_ (3.03 × 10^−4^ h^−1^) but with a conserved k_onRISC_ (2.73 × 10^−4^ h^−1^) to strive for the simplest scaling approach possible; however, the model could not readily describe the data using this approach, as depicted in Figure S6B—represented by the dashed lines. By optimizing the k_DR_ to a 5-fold lower value (6.41 × 10^−4^ h^−1^) and k_onRISC_ to a 10-fold higher value (2.26 × 10^−3^ h^−1^), the model could characterize the target protein knockdown with a 10–15% variation in the maximum knockdown, as shown in Figure S6B. Species-specific PBPK and PD effect parameters for Olpasiran© in monkey and human are shown in Table S1.

### 4.4. Sensitivity Analysis

**Figure 7:**
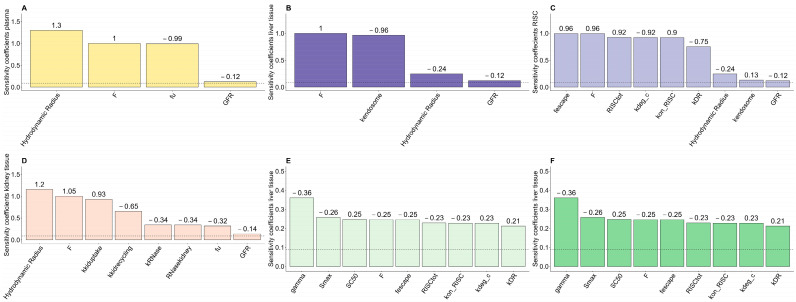
Model sensitivity analysis of PBPK parameters measured as the change in area under the curve (AUC) (ug·h/mL) simulated from time 0 h to time 8000 h (AUC_0-8000h_) for 1 mg/kg in (**A**) plasma, (**B**) liver tissue, (**C**) siRNA-induced RISC, (**D**) kidney tissue, (**E**) mRNA silencing, (**F**) downstream effect on target protein. A positive sensitivity coefficient indicates that a 10% increase in the investigated parameter leads to an increase in AUC_0-8000h_. Conversely, negative sensitivity coefficients indicate that a 10% increase in the investigated parameter results in a decrease in AUC_0-8000h_. The dashed line represents the threshold of 0.1, which is used to evaluate the minimum impact of the PBPK parameter.

The human model was subject to a local parameter sensitivity analysis performed towards total accumulation (AUC_0-8000h_) of GalNAc-siRNA in plasma, liver tissue, RISC formation, kidney tissue, mRNA silencing effect, and knockdown of target protein. The PK-PD relationship was not expected to show any dose-dependent non-linearity in the range of therapeutic doses (0.1–13 mg/kg); thus, the sensitivity analysis was conducted for a 1 mg/kg dose only. The plasma, liver tissue, RISC, and kidney tissue accumulation were mainly sensitive towards the hydrodynamic radius, F, and GFR (Figure 7A–D). The plasma and the kidney exposure also showed sensitivity towards the fu, and the kidney exposure further showed sensitivity towards kidney-specific parameters (k_kiduptake_, k_kidrecycling_, and RNase_kidney_) (Figure 7A,D). As expected from previous PBPK models, the liver exposure further showed high sensitivity towards k_endosome_ (Figure 7B). The RISC formation, mRNA silencing, and the downstream effect of target protein mainly showed sensitivity towards parameters directly associated with these processes (Figure 7C,E,F).

## 5. Discussion

GalNAc-siRNAs are an emerging class of drugs, and the necessity to understand their overall distribution and pharmacodynamics is an important aspect when establishing clinical PK-PD relationships. PBPK modeling is a valuable tool for not only mechanistically describing the mechanism of action but also quantifying the distribution of drugs into off-target tissues and supporting drug safety and dose assessments. Such capabilities are especially valuable in scenarios for humans when tissue data are generally limited compared to preclinical species [23]. In this study, a previously published WB-PBPK-PD model was further validated in mice using three internal Novo Nordisk compounds, then translated to monkeys and humans to quantify off-target tissue distribution and establish the clinical PK-PD relationship [11].

The initial phase of the investigation used three internal GalNAc-siRNAs with the same chemical stabilization design to minimize their influence on the results to facilitate clearer conclusions and interpretations. To further explore species-specific differences, establish the clinical PK-PD relationship, and enhance confidence in the model to serve as a framework for various GalNAc-siRNAs, a GalNAc-siRNA from the literature—Olpasiran©—was included in the analysis [13].

The current model confirmed compound-specific differences previously addressed by the legacy model, e.g., the bioavailability after subcutaneous administration. The subcutaneous bioavailability was estimated to be 22–47% for the included compounds, which is consistent with earlier reported values for GalNAc-siRNAs, 30% to 73% [11,15]. Furthermore, the established model revealed several previously unrecognized processes subject to species differences, such as subcutaneous absorption rate, unspecific liver permeability, as well as stability in and recycling from liver endosomes. A tendency of slower absorption, redistribution rate, endosomal degradation rate, and permeability was observed in humans compared to mice and monkey. The less-efficient endosomal degradation of GalNAc-siRNAs in the liver for humans is in line with the much longer duration of the pharmacological effect observed compared to the duration in rodents [5].

Overall, the simulated exposure for plasma and liver across species was within a 2-fold range of the observed exposure, and the model further met the criteria for successful model performance. The model also demonstrated that the plasma PK are highly conserved across compounds, which is consistent with previous findings from PK studies of various GalNAc-siRNAs in humans [24]. However, the model showed a tendency towards underpredicting liver tissue concentrations in mice (AFE = 0.78), while a trend for overprediction was seen in monkey (AFE = 1.32). A potential explanation for these results could be unaccounted species-specific variations to the subunit of the ASGPR [25]. Variation in these subunits may alter receptor dynamics with consequences for the GalNAc-siRNA’s liver distribution and the potential for cross-species translations [6,25]. An underprediction of exposure in the liver in the mouse model could potentially impact safety assessments when scaled into humans but since toxicities are rarely observed for GalNAc-siRNAs in general, the model is therefore considered safe to use as a guidance for novel GalNAc-siRNAs [26]. To the contrary, the overprediction in liver tissue in monkeys could theoretically lead to underdosing in clinical dose assessments since the pharmacological response of GalNAc-siRNAs is highly dose-dependent [27]. Nevertheless, the preclinical-to-clinical PK-PD relationship was successfully established across species and compounds by the adopted strategy of optimizing RISC model parameters (k_onRISC_ and k_DR_) while conserving PD effect parameters (S_max_ and SC_50_). We therefore consider the model adequate as a framework for clinical dose selection.

In the establishment of preclinical-to-clinical PK-PD relationship, some key aspects of consideration for future application of the model were identified. First, the association rate of the RNA-induced silencing complex (RISC) was found to be 16-fold slower in humans than in monkeys for the internal compounds and 8-fold higher in humans compared to monkey for Olpasiran©. Irrespective of the compound, the degradation rate of RISC was found to be 5–10-fold lower in humans compared to monkey. Comparable differences in RISC dynamics across compounds and species have been described in a previously published mechanistic mPBPK-PD model for GalNAc-siRNAs, as well as in the legacy model [11,16,28]. The variation in the association rate could potentially originate from species-specific differences in the gene expression of the argonaut 2 proteins or depend on the RNA sequence interactions with the Ago2 proteins [3,11,29]. It has also been suggested that variation in the RISC dynamics could originate from differences in metabolic stability and chemical stabilization design of the siRNA backbone and not solely on sequence-specific interactions with RNA machinery [30]. In PK validation, differences in intracellular metabolic stability were accounted for by estimating endosomal stability as a compound-specific parameter. However, the absence of liver data precluded identifications of Olpasiran’s endosomal stability and, consequently, comparisons with the differently stabilized siRNAs (siRNA-1, -2, and -3). Furthermore, these uncharacterized differences in their respective liver stabilities may also be a contributing factor to the observed variance in RISC dynamics between Olpasiran© and the in-house compounds. Besides the endosomal stability, it is also a possibility that the intracellular degradation in the cytoplasm also affects RISC formation and could be more appropriately modeled as a compound- and species-specific parameter, rather than being solely attributed to RISC association. In summary, these findings highlight the need for a deeper mechanistic understanding of RISC dynamics, as well as the importance of developing possibilities to inform these processes through in vitro measurements. Such understanding and data information would contribute significantly to further refinements of the species translation in the PK-PD relationship across different GalNAc-siRNAs and to improve the predictive capabilities of this model.

## 6. Conclusions

In this study, we successfully validated and scaled a previously established generic GalNAc-siRNA WB-PBPK-PD model for preclinical-to-clinical translation. The refined model effectively distinguishes between compound- and species-specific parameters, enabling the prediction of both on-target (liver) and off-target tissue concentrations. While this study confirmed that plasma PK are highly conserved across different siRNA compounds, it also revealed significant differences in their PK-PD relationships. This highlights a critical knowledge gap concerning the siRNA-Ago2 interaction, underscoring the need for further research and means to inform the model in this respect. Ultimately, this validated framework represents a valuable tool for the preclinical and clinical development of novel GalNAc-siRNA therapeutics, facilitating early assessments of efficacy and safety.

## Figures and Tables

**Table 1:** Summary of GalNAc-siRNAs included in model validation, species translation, and establishment of clinical PK-PD relationships.

Compound	Chemical Stabilization Design	Species	Dose Administration ^b^	Measurements ^c^	Modeling Steps	Reference
siRNA-2	Hairpin Loop	Mouse	3, 100, 300 mg/kg	Plasma/Liver/ Kidney/Gonads/Lung/Spleen	Validation of legacy model and off-target tissue PK	Internal Data
		Monkey	1 mg/kg	Plasma/Liver/ mRNA	Species translation	
		Human	0.1, 1, 3, 6, 12 mg/kg	Plasma/ Target protein	Species translation, clinical PK-PD	
siRNA-3	Hairpin Loop	Mouse	3, 100 mg/kg	Plasma/Liver/ Kidney	Validation of legacy model and off-target tissue PK	Internal Data
		Monkey	3 mg/kg	Plasma/Liver/ mRNA	Species translation	
		Human	1.5, 3, 6 mg/kg	Plasma/ Target protein	Species translation, clinical PK-PD	
siRNA-1	Hairpin Loop	Mouse	3, 10, 100 mg/kg	Plasma/Liver/ Kidney/Gonads/Lung/Spleen/ mRNA	Validation of legacy model and off-target tissue PK	Internal Data
		Monkey	3 mg/kg	Plasma/Liver/mRNA	Species translation	
		Human	1, 3.5, 6.5, 13 mg/kg	Plasma	Species translation, clinical PK-PD	
Olpasiran©	ECS ^a^	Monkey	10 mg/kg	Plasma/ Target protein	Evaluation of key considerations in clinical PK-PD, species translation, and RISC formation	Koren et al. 2022 [13]
		Human	3, 9, 30, 75, 225 mg			

a: Enhanced chemical stabilization design; b: values indicate subcutaneous doses (mg/kg or mg) in reference data; c: plasma and tissues indicate concentration measurements for PK assessments, mRNA and target protein indicate baseline normalized measurements in liver and plasma for PD assessments, respectively.

**Table 2:** Summary of global and species-specific PBPK model parameters.

Model Parameter (Unit)	Parameter Description	Mouse	Monkey	Human
ka (h^−1^)	Absorption rate constant	0.84 (Salim et al., 2025) [11].	4.57 (Optimized)	7.73 (Optimized)
P_liver_ (cm/min)	Endothelial permeability	0.02 (Salim et al., 2025) [11]	3.05 × 10^−3^ (Optimized)	1.21 × 10^−4^ (Optimized)
fu	Fraction of free GalNAc-siRNA in plasma	1.0 (Salim et al., 2025) [11].		
k_upatke.liver_ (min^−1^)	Liver endosomal uptake rate constant in remaining tissue	0.29 (Niederalt et al. 2018) [12]		
k_recycling.liver_ (min^−1^)	Liver endosomal recycling rate constant in remaining tissue	1.33 × 10^−3^ (Optimized)	8.22 × 10^−4^ (Optimized)	4.27 × 10^−5^ (Optimized)
k_kid.uptake_ (min^−1^)	Kidney endosomal uptake rate constant in remaining tissue	24.98 (Optimized)		
k_kid.recycling_ (min^−1^)	Kidney endosomal recycling rate constant in remaining tissue	3.90 × 10^−4^ (Salim et al., 2025) [11]		
k_gonads.uptake_ (min^−1^)	Gonad endosomal uptake rate constant in remaining tissue	1.48 (Optimized)		
k_lung.uptake_ (min^−1^)	Lung endosomal uptake rate constant in remaining tissue	0.06 (Optimized)		
K_heart.uptake_ (min^−1^)	Heart endosomal uptake rate constant in remaining tissue	0.39 (Optimized)		
k_spleen.uptake_ (min^−1^)	Spleen endosomal uptake rate constant in remaining tissue	0.17 (Optimized)		
k_RNase_ (h^−1^)	Ribonuclease degradation rate constant	1.21 × 10^−4^ (Salim et al., 2025) [11].		
RNase_kidney_ (μmol/l)	Ribonuclease concentration in kidney tissue	1.17 (Optimized)		
RNase_remaining_ (μmol/l)	Ribonuclease concentration in remaining tissue	2.75 × 10^−2^ (Optimized)		
R_tot_ (μmol/l)	Total ASGPR density	5.23 (Salim et al., 2025) [11].	2.46 ^a^	2.46 ^a^
k_on_ (l/nmol/h)	Association rate constant between GalNAc-siRNA and ASGPR	0.53 (Ayyar et al., 2021) [15].		
k_off_ (h^−1^)	Dissociation rate constant between GalNAc-siRNA and ASGPR	1.53 (Sato et al., 2002) [18].		
k_deg.R_ (h^−1^)	Degradation rate constant of ASGPR in cytoplasm	1.53 (Schwartz et al., 1982) [19].		
k_deg_ (h^−1^)	Degradation rate constant of ASGPR on hepatocyte	1.52 (Salim et al., 2025) [11].		
k_syn_ (h^−1^)	Synthesis rate constant of ASGPR	7.94 (k_syn_ = R_tot_ × k_deg_)		
k_int_ (h^−1^)	Internalization rate constant of GalNAc-siRNA-ASGPR complex	5.14 (Salim et al. 2025) [11].		
k_cle_ (h^−1^)	Cleavage rate constant of GalNAc-siRNA in liver endosome	1.32 (Prakash et al., 2014) [20].		
k_rec_ (h^−1^)	Recycling rate constant of ASGPR	13.8 (Schwartz et al., 1982) [19].		
k_endosome_ (h^−1^)	Liver endosomal degradation rate for siRNA	5.0 × 10^−3^ (Optimized)	−0.25 ^b^	−0.25 ^b^
f_escape_	Fraction of antisense strand escaping the liver endosome into cytoplasm	0.01 (McDougall et al. 2022) [5]		
k_deg.C_ (h^−1^)	siRNA degradation rate constant in cytoplasm	0.1 (Ayyar et al. 2021) [15].		
RISC_tot_ (umol/l)	Total RISC concentration	0.0003 (Wang et al., 2012) [21].		
k_onRISC_ (h^−1^)	Association rate constant of siRNA antisense strand and RISC	2.73 × 10^−4^ (Salim et al., 2025) [11]		1.68 × 10^−5^ (Optimized)
k_off.RISC_ (h^−1^)	Dissociation rate constant of siRNA antisense strand and RISC	1 × 10^−7^ (Barlett and Davis 2006) [22].		
S_max_	Maximum stimulation of mRNA degradation	58.84		
SC_50_ (nmol/L)	RISC-loaded siRNA at half-maximal stimulation	3.52		
k_deg.mRNA_ (h^−1^)	Degradation rate constant for mRNA	0.06 (Ayyar et al. 2021) [15].		
k_deg.protein_ (h^−1^)	Degradation rate target for protein	0.05 (Ayyar et al. 2021) [15].		
Gamma (γ)		1.5 (Ayyar et al. 2021) [15].		

a: See Ayyar et al. 2021 [15] for further information on receptor density scaling across species; b: allometric scaling exponent. See Equation (1) for calculation.

**Table 3:** Summary of compound-specific PBPK model parameters.

Parameter (Unit)	Parameter Description	Compound	Species	Value
F (%)	Bioavailability	siRNA-1	Mouse	47
			Monkey	
			Human	
		siRNA-2	Mouse	22
			Monkey	
			Human	
		siRNA-3	Mouse	
			Monkey	
			Human	
k_recycle.tissue_ (min^−1^)	Endosomal recycling rate constant in remaining tissue	siRNA-1	Mouse	3.23 × 10^−5^
			Monkey	
			Human	
		siRNA-2	Mouse	3.49 × 10^−4^
			Monkey	
			Human	
		siRNA-3	Mouse	3.23 × 10^−5^
			Monkey	
			Human	
k_DR_ (h^−1^)	Degradation rate constant of RISC complex	siRNA-1	Mouse	9.72 × 10^−3^
			Monkey	8.02 × 10^−3^
			Human	-
		siRNA-2	Mouse	-
			Monkey	5.28 × 10^−3^
			Human	4.17 × 10^−4^
		siRNA-3	Mouse	-
			Monkey	1.94 × 10^−3^
			Human	2.02 × 10^−4^

## Data Availability

The data presented in this study are available in this article and the Supplementary Materials.

## Supplementary Materials

The following supporting information can be downloaded at https://www.mdpi.com/article/10.3390/pharmaceutics17091154/s1, Figure S1: Graphic illustration of the whole body PBPK-PD model structure. Figure S2: Schematic representation of the model development for GalNAc-siRNAs. The flow chart describes the different modelling steps: Model validation, species translation, establishment of clinical PK-PD relationship and key considerations of RISC formation. Figure S3: Model-simulated kidney tissue concentration profiles vs. observed data for subcutaneous (SC)-administered GalNAc-conjugated siRNA-1, siRNA-2, and siRNA-3 in mouse. Figure S4: Model-simulated knockdown of target mRNA vs. observed data for subcutaneous (SC)-administered GalNAc-conjugated siRNA-1 in mouse; Figure S5: Model-simulated plasma concentration profiles and downstream effect of target protein vs. observed data (Koren et al., 2022) [13] for subcutaneous (SC)-administered GalNAc-conjugated siRNA, Olpasiran© 10 mg/kg SC in monkey; Figure S6: Model-simulated plasma concentration profiles and downstream effect of target protein vs. observed data (Koren et al., 2022) [13] for subcutaneous (SC)-administered GalNAc-conjugated siRNA, Olpasiran© in human, Figure S7: Overview of the calculated average fold error (AFE) and absolute average fold error (AAFE) of the geometric mean of the simulated AUC_sim_ and observed AUC_obs_. Table S1: Summary of compound- and species-specific PBPK model parameters for Olpasiran© in monkey and human; Table S2: Summary of the observed area under the curve (AUC_obs_) and simulated area under the curve (AUC_sim_) for each dose/compound and the AUC_sim_/AUC_obs_ ratios given as the fold change for each dose/compound of plasma concentrations in the mouse model; Table S3: Summary of the observed area under the curve (AUC_obs_) and simulated area under the curve (AUC_sim_) for each dose/compound and the AUC_sim_/AUC_obs_ ratios given as the fold change for each dose/compound of plasma concentrations in the human model; Table S4: Summary of the observed area under the curve (AUC_obs_) and simulated area under the curve (AUC_sim_) for each dose/compound and the AUC_sim_/AUC_obs_ ratios given as the fold change for each dose/compound of plasma concentrations in the human model. Supplementary Model File: Case_Example_WB-PBPK-PD_Model_Human_3_mgkg_siRNA-2.
