# Supplementary material for: Whole-Body Physiologically Based Pharmacokinetic–Pharmacodynamic Modeling for Interspecies Translation and Mechanistic Characterization of Plasma and Tissue Disposition of GalNAc-siRNAs
Source: Pharmaceutics. 2025 Sep 3;17(9):1154. doi: 10.3390/pharmaceutics17091154 (PMC12472881; doi:10.3390/pharmaceutics17091154)
Supplement: Supplementary file 1 [file pharmaceutics-17-01154-s001.zip › pharmaceutics-3818745-supplementary/pharmaceutics-3818745-supplementary.pdf]

Article

Whole-Body Physiologically Based Pharmacokinetic–Pharmacodynamic Modeling for Interspecies Translation and Mechanistic Characterization of Plasma and Tissue Disposition of GalNAc-siRNAs

Emilie Langeskov Salim [1,2], Kim Kristensen [2], Girish Chopda [3] and Erik Sjögren [1,*]

[1] Department of Pharmaceutical Bioscience, Translational Drug Discovery and Development, Uppsala University, SE-75124 Uppsala, Sweden; emlv@novonordisk.com

[2] Department of Discovery PKPD & QSP Modelling, Novo Nordisk A/S, DK-2760 Måløv, Denmark; kkri@novonordisk.com

[3] Department of Nonclinical and Clinical Pharmacology, Novo Nordisk, 800 Scudders Mill Road, Plainsboro, Boston, NJ 08536, USA; gpcp@novonordisk.com

* Correspondence: erik.sjogren@uu.se; Tel.: +46-18-471-41-54

Supplementary

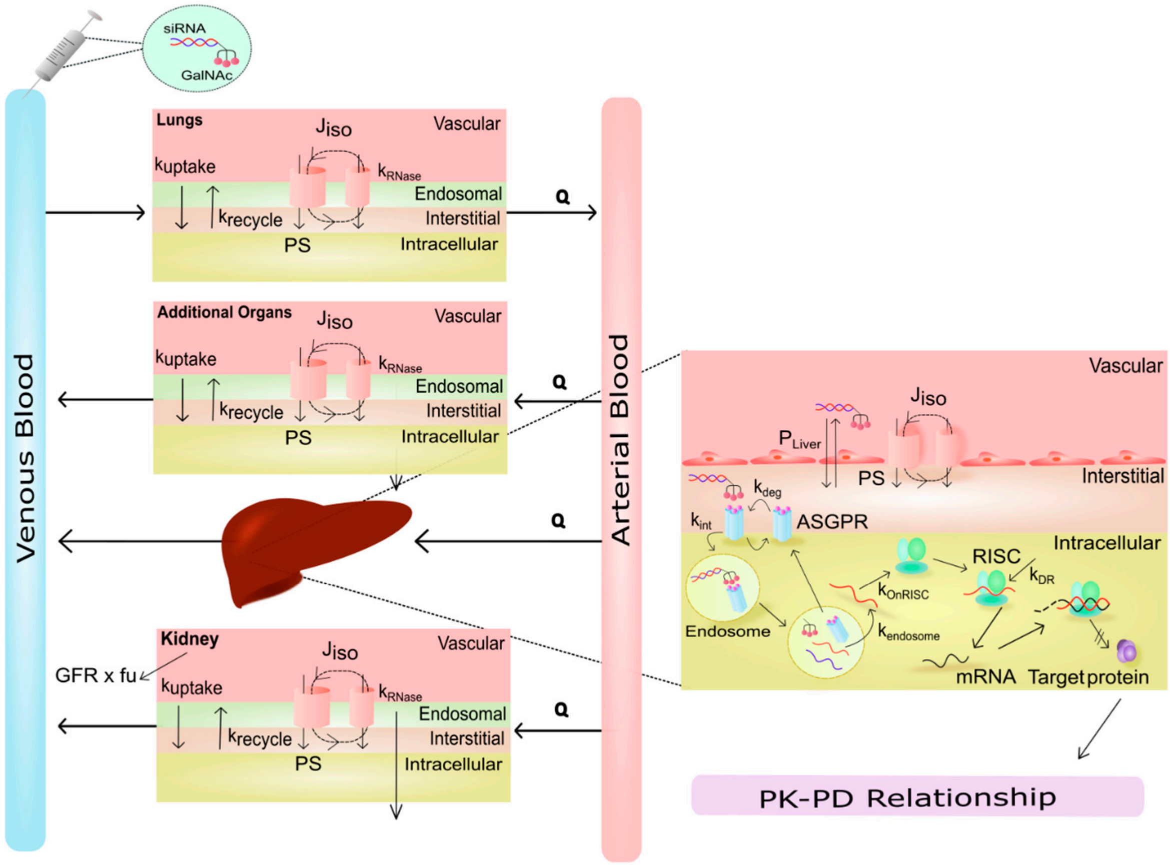

Figure S1. Graphic illustration of the whole body PBPK-PD model structure. Left panel illustrates the generic structure of a whole body PBPK model for GalNAc-siRNAs with the two-pore formalism characterizing the tissue extravasation. Right panel shows the liver dynamics including the two-pore formalism for extravasation as well as the bidirectional liver permeability.

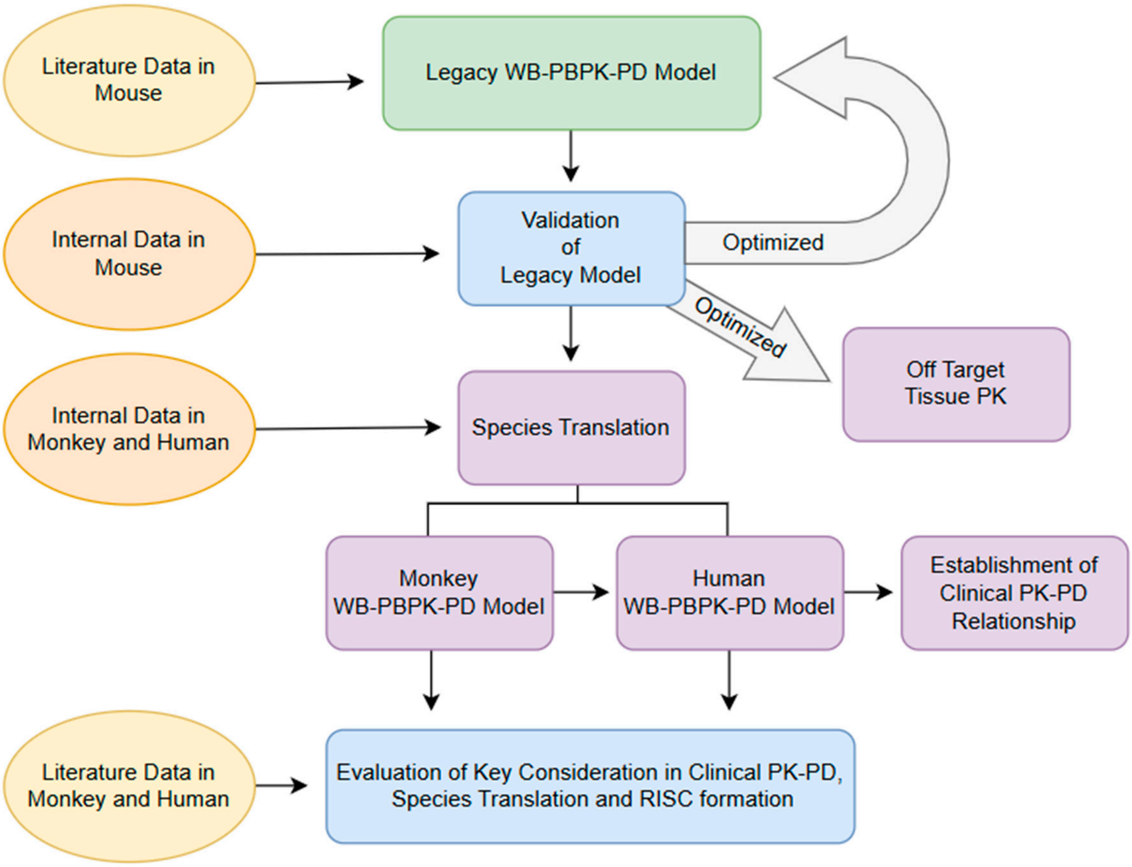

Figure S2. Schematic representation of the model development for GalNAc-siRNAs. The flow chart describes the different modelling steps: Model validation, species translation, establishment of clinical PK-PD relationship and key considerations of RISC formation. .

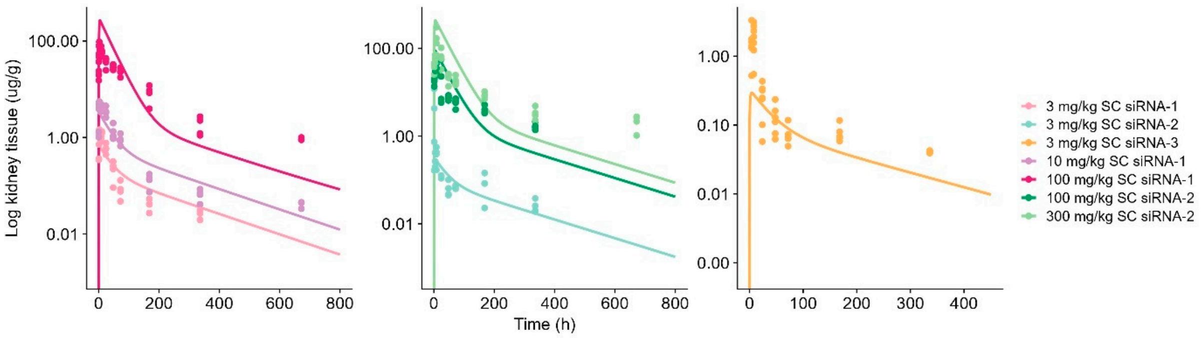

Figure S3. Model-simulated kidney tissue concentration profiles vs. observed data for subcutaneous (SC) administered GalNAc conjugated siRNA-1, siRNA-2 and siRNA-3 in mouse. Solid line represents model simulated concentrations and dots represent observed data. Left panel shows data on siRNA-1 after 3 mg/kg (light pink), 10 mg/kg (light purple) and 100 mg/kg (dark pink). Mid panel shows siRNA 2 after 3 mg/kg (light blue), 100 mg/kg (dark green) and 300 mg/kg (light green). Right panel shows siRNA-3 after 3 mg/kg (light orange). .

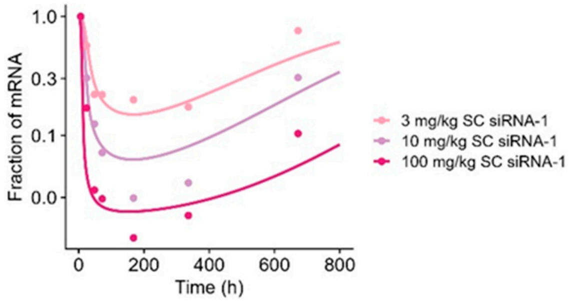

Figure S4. Model-simulated knockdown of target mRNA vs. observed data for subcutaneous (SC) administered GalNAc conjugated siRNA-1 in mouse. Solid line represents model simulated concentrations and dots represent observed data. Light pink line represents siRNA-1, 3 mg/kg (SC). Purple line represents siRNA-1, 10 mg/kg (SC). Dark pink line represents siRNA-1, 100 mg/kg (SC).

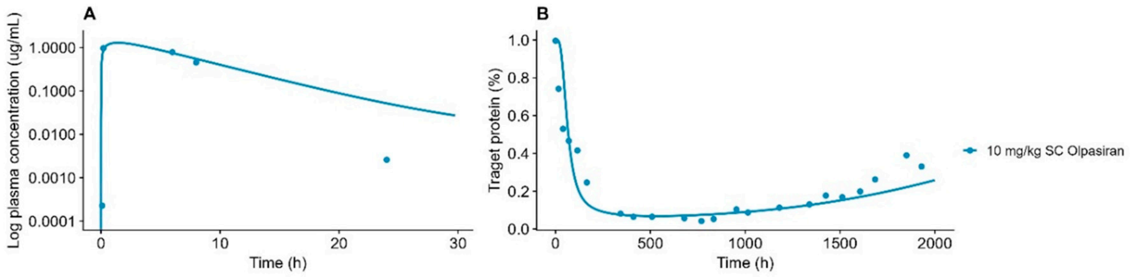

Figure S5. Model-simulated plasma concentration profiles and downstream effect of target protein vs. observed data (Koren et al., 2022) for subcutaneous (SC) administered GalNAc conjugated siRNA, Olpasiran© 10 mg/kg SC in monkey. In panel A the solid line represents model simulated plasma concentrations based on PBPK parameters retrieved from siRNA-1, siRNA-2 and siRNA-3 and dots represent observed data. In panel B the solid line represents model simulated downstream effect on target protein with optimized RISC PK and PD effect parameters (kDR, konRISC, Smax and SC50) and dots represent observed data. Light blue line represents Olpasiran©, 3 mg (SC).

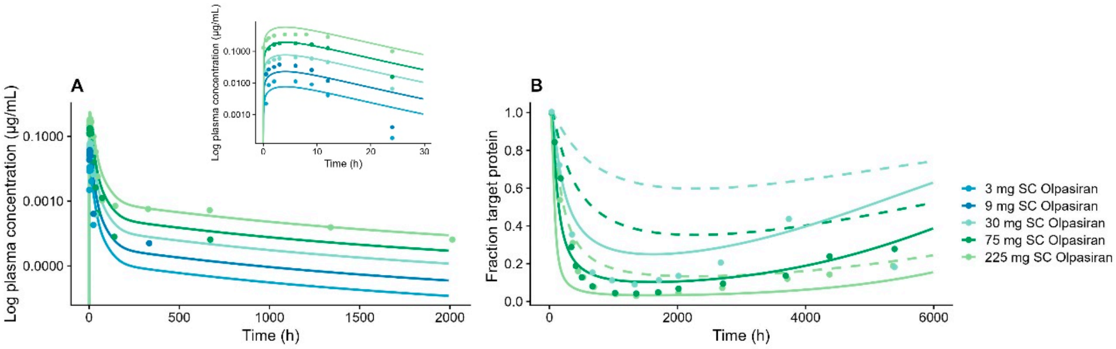

Figure S6. Model-simulated plasma concentration profiles and downstream effect of target protein vs. observed data (Koren et al., 2022) for subcutaneous (SC) administered GalNAc conjugated siRNA, Olpasiran© in human. In panel A the solid line represents model simulated plasma concentrations based on PBPK parameters retrieved from siRNA-3 and dots represent observed data. In panel B the solid line represents model simulated downstream effect on target protein with optimized RISC PK parameters (kDR and konRISC). In panel B dashed line represents model simulated downstream effect on target protein with kDR being fixed to a 10-fold lower value compared to estimates retrieved in monkey model. Light blue line represents Olpasiran©, 3 mg (SC). Dark blue line represents Olpasiran©, 9 mg (SC). Light green line represents Olpasiran©, 30 mg (SC). Dark green line represents Olpasiran©, 75 mg (SC). Light green line represents Olpasiran©.

Table S1. Summary of compound and species-specific PBPK model parameters for Olpasiran© in monkey and human.

Model Parameter (Unit)	Parameter description	Monkey	Human
**F (%)**	Absorption rate constant	22[a]	
**$k_{endosome}$ (h^{-1})**	Liver endosomal degradation rate for siRNA	-0.25[a,b]	-0.25[a,b]
**$k_{recycle}$ (min^{-1})**	Endosomal recycling rate constant in remaining tissue	$3.23 \cdot 10^{-5}$[a]	
**k_{onRISC} (h/nmol/l)**	Association rate constant of siRNA antisense strand and RISC	$2.73 \cdot 10^{-4}$ Salim et al., 2025	$2.26 \cdot 10^{-3}$ (Optimized)
**k_{DR} (h^{-1})**	Degradation rate constant of RISC complex	$3.03 \cdot 10^{-3}$ (Optimized)	$6.41 \cdot 10^{-4}$ (Optimized)
**S_{max}**	Maximum stimulation of mRNA degradation	36.4 (Optimized)	
**SC_{50} (nmol/l)**	RISC loaded siRNA at half maximal stimulation	8.61 (Optimized)	
**$k_{deg.mRNA}$ (h^{-1})**	Degradation rate constant for mRNA	0.06 (Ayyar et al. 2021)	
**$k_{deg.protein}$ (h^{-1})**	Degradation rate target for protein	0.05 (Ayyar et al. 2021)	
**Gamma (γ)**	Gamma coefficient for target protein knockdown	1.5 (Fixed)	

a: Compound specific model parameter retrieved from siRNA-3; b: Allometric scaling exponent.

Model Validation

The AUC_{obs} was compared to the AUC_{sim} by calculating the fold change given as the ratio between AUC_{obs} and AUC_{sim} as described in equation S1:

$$\text{Fold Change} = \frac{AUC_{sim}}{AUC_{obs}} \quad \text{(S1)}$$

Table S2 Summary of the observed area under the curve (AUC_{obs}) and simulated area under the curve (AUC_{sim}) for each dose/compound and the AUC_{sim}/AUC_{obs} ratios given as the fold change for each dose/compound of plasma concentrations in the mouse model.

Dose/Compound	Measurement	AUC_{sim} (ug·h/mL)	AUC_{obs} (ug·h/mL)	Fold Change (AUC_{sim}/AUC_{obs})
3 mg/kg siRNA-1	Plasma	0.42	0.64	0.66
3 mg/kg siRNA-2	Plasma	0.18	0.13	1.38
3 mg/kg siRNA-3	Plasma	0.18	0.15	1.19
10 mg/kg siRNA-1	Plasma	3.93	3.39	1.16
100 mg/kg siRNA-1	Plasma	276	128	2.15
100 mg/kg siRNA-2	Plasma	92.53	49.3	1.88
100 mg/kg siRNA-3	Plasma	89.7	43.7	2.05
300 mg/kg siRNA-2	Plasma	446	207	2.15
3 mg/kg siRNA-1	Liver	4478	3322	1.35
3 mg/kg siRNA-2	Liver	2154	2697	0.80
3 mg/kg siRNA-3	Liver	2151	3295	0.65
10 mg/kg siRNA-1	Liver	14655	16116	0.91
100 mg/kg siRNA-1	Liver	79019	105994	0.75
100 mg/kg siRNA-2	Liver	49800	51521	0.97
100 mg/kg siRNA-3	Liver	49321	108510	0.45
300 mg/kg siRNA-2	Liver	100933	158213	0.64
3 mg/kg siRNA-1	Kidney	38.7	46.3	0.84
3 mg/kg siRNA-2	Kidney	18.5	28.7	0.65
3 mg/kg siRNA-3	Kidney	18.5	43.9	0.42
10 mg/kg siRNA-1	Kidney	156	215.7	0.72
100 mg/kg siRNA-1	Kidney	8312	4648	1.79
100 mg/kg siRNA-2	Kidney	2857	2068	1.38
300 mg/kg siRNA-2	Kidney	13626	6830	2.00

Table S3. Summary of the observed area under the curve (AUC_{obs}) and simulated area under the curve (AUC_{sim}) for each dose/compound and the AUC_{sim}/AUC_{obs} ratios given as the fold change for each dose/compound of plasma concentrations in the human model.

Dose/Compound	Measurement	AUCsim (ug·h/mL)	AUC_{obs} (ug·h/mL)	Fold Change (AUC_{sim}/AUC_{obs})
1 mg/kg siRNA-2	Plasma	1.01	1.87	0.54
1.5 mg/kg siRNA-1	Plasma	2.10	2.13	1.39
3 mg/kg siRNA-1	Plasma	5.77	6.56	0.88
3 mg/kg siRNA-3	Plasma	2.78	3.59	0.77
1 mg/kg siRNA-2	Liver	6911	7901	0.87
1.5 mg/kg siRNA-1	Liver	22938	15496	1.48
3 mg/kg siRNA-1	Liver	45872	31778	1.44
3 mg/kg siRNA-3	Liver	16521	10073	1.64

Table S4. Summary of the observed area under the curve (AUC_{obs}) and simulated area under the curve (AUC_{sim}) for each dose/compound and the AUC_{sim}/AUC_{obs} ratios given as the fold change for each dose/compound of plasma concentrations in the human model.

Dose/Compound	Measurement	AUC_{sim} (ug·h/mL)	AUC_{obs} (ug·h/mL)	Fold Change (AUC_{sim}/AUC_{obs})
0.1 mg/kg siRNA-2	Plasma	0.23	0.34	0.66
0.3 mg/kg siRNA-3	Plasma	0.76	1.04	0.74
1 mg/kg siRNA-1	Plasma	5.40	6.54	0.83
1 mg/kg siRNA-2	Plasma	2.65	4.05	0.65
1.5 mg/kg siRNA-3	Plasma	3.80	7.60	0.50
3 mg/kg siRNA-2	Plasma	8.27	12.2	0.68
3 mg/kg siRNA-3	Plasma	7.61	18.63	0.41
3.5 mg/kg siRNA-1	Plasma	16.69	18.6	0.90
6 mg/kg siRNA-2	Plasma	16.54	28.5	0.58
6 mg/kg siRNA-3	Plasma	15.2	27.4	0.55
6.5 mg/kg siRNA-1	Plasma	35.27	46.3	0.76
12 mg/kg siRNA-2	Plasma	33.1	67.3	0.49
12 mg/kg siRNA-3	Plasma	30.5	71.29	0.43
13 mg/kg siRNA-1	Plasma	75.8	109	0.69

The model was additionally assessed quantitatively by calculating the average fold error (AFE) and the absolute average fold error (AAFE) for each measurement, as outlined in equations S2 and S3:

$$\text{average fold error} = 10^{\sum \log\left(\frac{AUC_{sim}}{AUC_{obs}}\right)/n} \quad (S2)$$

$$\text{absolute average fold error} = 10^{\sum \left|\log\left(\frac{AUC_{sim}}{AUC_{obs}}\right)\right|/n} \quad (S3)$$

Where AUC_{sim} is the simulated AUC; AUC_{obs} is the observed AUC; n is the number of observations of the respective measurement.

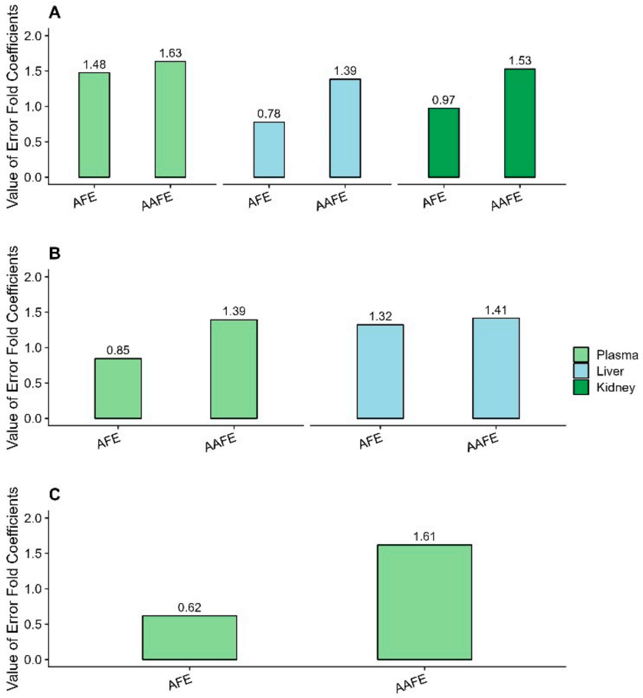

Figure S7. Overview of the calculated average fold error (AFE) and absolute average fold error (AAFE) of the geometric mean of the simulated AUC_{sim} and observed AUC_{obs} in A) mouse model, B) monkey model, C) human model with each available measurement depicted as plasma (light green color), liver (light blue color and kidney (dark green color) .
